# Complete genome sequence of *Pseudomonas citronellolis* P3B5, a candidate for microbial phyllo-remediation of hydrocarbon-contaminated sites

**DOI:** 10.1186/s40793-016-0190-6

**Published:** 2016-09-26

**Authors:** Mitja N.P. Remus-Emsermann, Michael Schmid, Maria-Theresia Gekenidis, Cosima Pelludat, Jürg E. Frey, Christian H. Ahrens, David Drissner

**Affiliations:** 1 Agroscope, Institute for Food Sciences IFS, Wädenswil, Switzerland; 2 Agroscope, Institute for Plant Production Sciences IPS, Wädenswil, Switzerland; 3 Swiss Institute of Bioinformatics, Wädenswil, Switzerland; 4 ETH Zurich, Institute of Food, Nutrition and Health, Zurich, Switzerland

**Keywords:** Phyllosphere, Complete genome, Pseudomonad, PacBio, Nonhybrid *de novo* assembly, Alkane degradation, Terpenoid degradation, Bioremediation potential

## Abstract

*Pseudomonas citronellolis* is a Gram negative, motile gammaproteobacterium belonging to the order *Pseudomonadales* and the family *Pseudomonadaceae*. We isolated strain P3B5 from the phyllosphere of basil plants (*Ocimum basilicum* L.). Here we describe the physiology of this microorganism, its full genome sequence, and detailed annotation. The 6.95 Mbp genome contains 6071 predicted protein coding sequences and 96 RNA coding sequences. *P. citronellolis* has been the subject of many studies including the investigation of long-chain aliphatic compounds and terpene degradation. Plant leaves are covered by long-chain aliphates making up a waxy layer that is associated with the leaf cuticle. In addition, basil leaves are known to contain high amounts of terpenoid substances, hinting to a potential nutrient niche that might be exploited by *P. citronellolis*. Furthermore, the isolated strain exhibited resistance to several antibiotics. To evaluate the potential of this strain as source of transferable antibiotic resistance genes on raw consumed herbs we therefore investigated if those resistances are encoded on mobile genetic elements. The availability of the genome will be helpful for comparative genomics of the phylogenetically broad pseudomonads, in particular with the sequence of the *P. citronellolis* type strain PRJDB205 not yet publicly available. The genome is discussed with respect to a phyllosphere related lifestyle, aliphate and terpenoid degradation, and antibiotic resistance.

**Electronic supplementary material:**

The online version of this article (doi:10.1186/s40793-016-0190-6) contains supplementary material, which is available to authorized users.

## Introduction

The genus *Pseudomonas* encompasses a large group of bacteria that are ecologically and functionally very diverse including many human and plant pathogenic species [1], but also species with mutualistic host interactions, the most prominent examples of which are biocontrol strains that protect plants from pathogens [2–4]. Due to their versatile properties and pathogenicity, pseudomonads garnered much attention in recent years and members of the genus have been the subject of full genome sequencing projects, i.e. at the time of writing the *Pseudomonas* Genome Database contained 98 complete genomes and 1447 draft genomes [5]. Up till now, this database is heavily biased towards pathogenic species, most prominently including 996 human-pathogenic *P. aeruginosa* strains and 105 plant pathogenic *P. syringae* strains. Taking into account the assigned species of the sequenced strains only, roughly a third (76 of 199) of the validly published *Pseudomonas* species [6] have been sequenced. Thus, a comprehensive picture of genetic diversity within the pseudomonads is lacking and investigating thus far unsequenced species will fill this knowledge gap.

*Pseudomonas citronellolis* has long been recognized for its ability to degrade hydrocarbons such as isoprenoid compounds including citronellol, hence its name [7], and complex oily sludge contaminations [8], indicating the species might potentially serve as bioremediation organism by cleaning up oil contaminated sites. The described strain was isolated from plant leaf material. Interestingly, plant leaves are covered by a cuticle consisting of cutin, an esterified aliphatic polymer [9] that is impregnated and overlaid by intra- and epi-cuticular waxes consisting of very long-chain aliphatic compounds, including alkanes, alcohols, and fatty acids [10]. The ability of *P. citronellolis* to degrade aliphatic compounds leads to the intriguing possibility that the bacterium may be able to exploit aliphatic compounds as a nutrient source during leaf surface colonization. Furthermore, the ability of *P. citronellolis* to also degrade terpenoid compounds [11] is another interesting feature that might enable this species to degrade the abundantly available terpene compounds from herbal plants [12] and hence may represent a growth advantage over other phyllosphere bacteria on such hosts.

*P. citronellolis* has previously been used as a model organism to investigate metabolic pathways and enzyme activity, such as glucose catabolism and gluconeogenesis [13], acyl-coenzyme A carboxylases [14], and terpene degradation [11]. The complete genome of *P. citronellolis* described here represents a useful resource for the ongoing environmental and physiological research in this species, and can serve as a starting point for detailed systems biology studies [15], at least until the genome sequence of the type strain PRJDB205 becomes available. We present a summary, classification and general physiological features of the strain *P. citronellolis* P3B5, as well as the genomic sequence, assembly, annotation, and its putative adaptations to a phyllosphere lifestyle.

## Organism information

### Classification and features

**Fig. 1:**
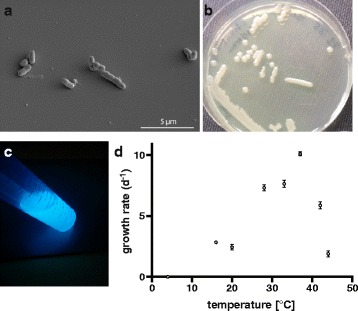
**a** Scanning-electron micrograph of *P. citronellolis* P3B5. **b** *P. citronellolis* P3B5 grown on LB agar for 4 days. **c** *P. citronellolis* P3B5 grown in M9 minimal medium for 20 h excited by UV light exhibiting strong fluorescence. **d** Growth of *P. citronellolis* P3B5 was analyzed by measuring the optical density at 600 nm at the different temperatures for 24 h. 12 to 15 replicate measurements were performed for each temperature. By plotting the observed growth rate during the exponential growth phase at different temperatures, it was determined that the ideal growth temperature of *P. citronellolis* P3B5 is around 37 °C. No growth was observed at 4 °C

The species *P. citronellolis* was proposed and described by Seubert [7] and isolated from soil collected under pine trees in northern Virginia, USA. *P. citronellolis* is a Gram negative, rod-shaped, gammaproteobacterium that is approximately 2 μm in length and 0.5 μm in width (Fig. 1a), motile by one polar flagellum, and non-spore-forming [7]. On Lysogeny Broth agar *P. citronellolis* forms white, round colonies (Fig. 1b), that produce green fluorescent pigments after several days of incubation. Grown in liquid M9 minimal medium the production of green fluorescent pigments is even more pronounced (Fig. 1c).

The here-described *P. citronellolis* P3B5 was recovered from healthy basil leaves. The species was initially identified by MALDI biotyping using a MicroFlex MALDI-TOF mass spectrometer, and the MALDI Biotyper DB V4.0.0.1 (Bruker Daltonics, Germany). MALDI biotyping has been shown to be able to identify pseudomonads at the species level with high accuracy [16, 17].

To isolate bacteria from the basil phyllosphere, 30 g of plant material were suspended in 100 ml peptone water (9 g/L NaCl, 1 g/L tryptone (Merck, Germany)), processed for 3 min in a stomacher (Smasher, AES Chemunex, France) and 100 μL aliquots of the resulting supernatant were plated onto different media, including the *Escherichia coli* selective TBX agar (Oxoid, UK). *P. citronellolis* P3B5 was isolated from the background microbiota on TBX agar, i.e. non-target bacteria growing on the agar, indicating that *P. citronellolis* utilizes similar compounds as *E. coli*. Furthermore, since the incubation took place at 44 °C, it is able to grow at high temperatures. However, the characteristic blue color indicative for *E. coli* colonies on TBX agar was not formed by *P. citronellolis*, indicating the absence of a functional β-glucuronidase in its genome.

During the survey not only *P. citronellolis* P3B5, but additional, not further characterized *P. citronellolis* isolates were frequently detected in marjoram and basil phyllosphere samples, implying *P. citronellolis* to be part of the plants indigenous phyllosphere communities. Pseudomonads are common colonizers of the phyllosphere [18] and can contribute to large proportions of its bacterial community [19–21].

**Fig. 2:**
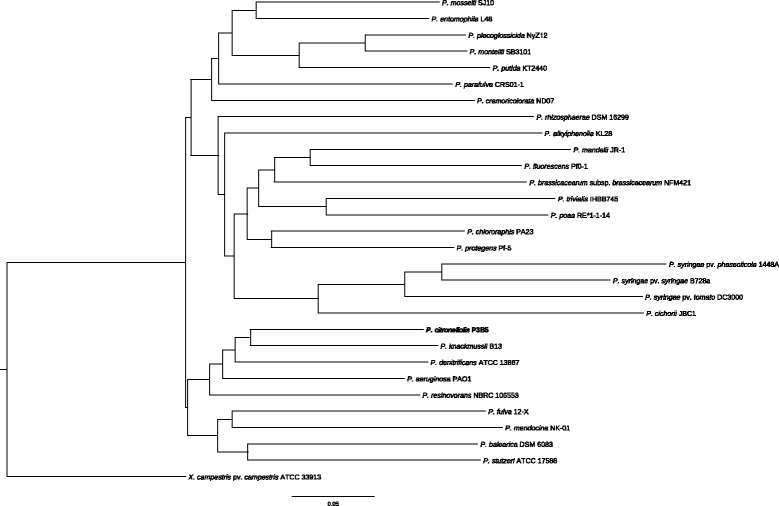
Phylogenetic tree of the genus *Pseudomonas* highlighting the position of *P. citronellolis* P3B5 relative to other representative *Pseudomonas* species. *Xanthomonas campestris* pv. *campestris* ATCC 33913 was chosen as outgroup. The tree is based on whole genome alignment. The bar reflects normalized pairwise genomic distance between genomes based on their shared genomic content. Accession numbers of the used strains are reported in Additional file 1: Table S1. The overall topology is confirmed by a phylogenetic tree based on MLSA with good bootstrap support (Additional file 2: Figure S2)

To phylogenetically place *P. citronellolis* P3B5, a phylogenetic tree was constructed by multiple genome alignment using the program progressiveMauve with standard parameters [22] (Fig. 2). From each thus far sequenced species one representative strain, if possible a type strain, was chosen. Only complete genomes were considered. As outgroup species *Xanthomonas campestris* pv. *campestris*ATCC 33913 was chosen. A summary of the classification and general features of *P. citronellolis* P3B5 is given in Table 1.

**Table 1:** Classification and general features of *P. citronellolis* P3B5 [30]

MIGS ID	Property	Term	Evidence code^a^
	Classification	Domain *Bacteria*	TAS [62]
		Phylum *Proteobacteria*	TAS [63]
		Class *Gammaproteobacteria*	TAS [64]
		Order *Pseudomonadales*	TAS [65, 66]
		Family *Pseudomonadaceae*	TAS [66, 67]
		Genus *Pseudomonas*	TAS [66, 68]
		Species *Pseudomonas citronellolis*	TAS [7, 23, 66]
		Type strain LMG 21218	TAS [7]
	Gram stain	Negative	TAS [7]
	Cell shape	Rod	TAS [7]
	Motility	Motile	TAS [7]
	Sporulation	Not reported	TAS [7]
	Temperature range	18–42 °C	IDA
	Optimum temperature	37 °C	IDA
	pH range; Optimum	not determined	IDA
	Carbon source	See paragraph “biochemical profiling”	IDA
MIGS-6	Habitat	Soil, phyllosphere	TAS [7], IDA
MIGS-6.3	Salinity	1–5 % NaCl (w/v)	IDA
MIGS-22	Oxygen requirement	Aerobic	TAS [7]
MIGS-15	Biotic relationship	Free living, symbiont	TAS [7], IDA
MIGS-14	Pathogenicity	Non-pathogen	NAS
MIGS-4	Geographic location	Switzerland/Zurich area	IDA
MIGS-5	Sample collection	02.07.2015	IDA
MIGS-4.1	Latitude	47°45′37 N	IDA
MIGS-4.2	Longitude	8°4′37 E	IDA
MIGS-4.4	Altitude	521 m	IDA

^a^ Evidence codes–*IDA* Inferred from Direct Assay, *TAS* Traceable Author Statement (i.e., a direct report exists in the literature), *NAS* Non-traceable Author Statement (i.e., not directly observed for the living, isolated sample, but based on a generally accepted property for the species, or anecdotal evidence). These evidence codes are from the Gene Ontology project [69]

### Biochemical profiling

To perform a detailed biochemical characterization of *P. citronellolis* P3B5, it was cultivated overnight in 25 mL MM2 medium containing l-asparagin and sorbitol (4 g l^−1^ L-asparagine, 2 g l^−1^ K_2_HPO_4_, 0.2 g l^−1^ MgSO_4_, 3 g l^−1^ NaCl, 10 g l^−1^ sorbitol) at 28 °C and 240 rpm, before it was harvested by centrifugation at 3500 × g for 10 min. The harvested cells were washed thrice in 1 × PBS buffer (8 g l^−1^ NaCl, 0.2 g l^−1^ KCl, 1.44 g l^−1^ Na_2_HPO_4_, 0.24 g l^−1^ KH_2_PO_4_, pH 7), before they were resuspended and diluted in 1 × PBS to reach an optical density of OD_600nm_ = 0.1. This suspension was then used for inoculation of Biolog GN2, GenIII, and AN plates (Biolog Inc, USA), which were incubated for several days at 28 °C and analyzed for changes of their optical density at 590 nm using a microtiter plate reader (Infinite M200, Tecan, Switzerland).

Analysis of the GN2 plates revealed that *P. citronellolis* P3B5 was able to utilize the following wide range of substrates: 2-aminoethanol, α-ketoglutaric acid, α-d-glucose, β-hydroxy butyric acid, bromo succinic acid, cis-aconitic acid, citric acid, d-alanine, d-cellobiose, d-fructose, d-galactonic acid lactone, d-galacturonic acid, d-gluconic acid, d-mannitol, d-mannose, d-melibiose, d-raffinose, d,l-α-glycerol phosphate, d,l-lactic acid, γ-amino butyric acid, glucose-1-phosphate, glucose-6-phosphate, hydroxy-l-proline, inosine, itaconic acid, l-alanine, l-arabinose, l-asparagine, l-aspartic acid, l-glutamic acid, l-histidine, l-proline, l-pyroglutamic acid, methyl pyruvate, mono-methyl-succinate, p-hydroxy phenylacetic acid, propionic acid, putrescine, quinic acid, sebacic acid, succinamic acid, succinic acid, sucrose, tween 40, tween 80, and urocanic acid. On Biolog GenIII plates, the following additional substrates were utilized: Acetic acid, α-hydroxy-butyric acid, α-ketobutyric acid, d-fructose-6-phosphate, d-galactose, d-glucose-6-phosphate, d-glucuronic acid, d-malic acid, d-maltose, d-trehalose, formic acid, l-arginine, l-galactonic acid lactone, l-lactic acid, l-malic acid, *N*-acetyl-d-neuramic acid, and pectin.

In contrast to previously described *P. citronellolis* strains, *P. citronellolis* P3B5 could not utilize d-mannitol, glycerol, d,l-carnitine, d-psicose, l-alanyl-glycine, and formic acid. However, compared to the previously described *P. citronellolis* strains, *P. citronellolis* P3B5 additionally utilized α-d-lactose, d-galactose, d-glucose-6-phosphate, d-glucuronic acid, d-maltose, d-mannitol, d-melibiose, d-raffinose, d-saccharic acid, d-trehalose, d,l-α-glycerol phosphate, glucose-1-phosphate, glucose-6-phosphate, hydroxy-l-proline, inosine, l-arabinose, l-galactonic acid lactone, *N*-acetyl-d-glucosamine, sucrose, and thymidine [7, 23]. The following compounds, that have not been tested in previous studies, were also utilized by *P. citronellolis* P3B5: α-hydroxy-butyric acid, α-ketobutyric acid, d-fructose-6-phosphate, d-mannose, fumaric acid, l-alanyl-l-histidine, l-alanyl-l-glutamine, l-glutamine, l-lactic acid, l-pyroglutamic acid, l-rhamnose, l-valine plus l-aspartic acid, *N*-acetyl-d-neuramic acid, p-hydroxy phenylacetic acid, pyruvic acid, quinic acid, and succinamic acid. In summary, based on the tested set of substrates, *P. citronellolis* P3B5 appears to be metabolically more versatile than previously isolated strains, which might reflect an adaptation to the phyllosphere environment.

### Growth of *P. citronellolis* P3B5 at different temperatures

*P. citronellolis* was incubated in 12 to 15 replicates each at 44, 42, 37, 33, 28, 20, and 16 °C in tryptic soy broth (Oxoid, UK) in a Bioscreen C MBR microwell growth analysis platform (Oy Growth Curves Ab Ltd, Finland). Absorbance at OD_600nm_ was determined every 30 min for a total of 24 h and the specific growth rate of the strain was derived from the exponential growth phase of the cultures. Growth rate at 4 °C was estimated by incubating a 200 mL shake flask filled with 50 mL tryptic soy broth in a cold room for several days and measuring absorbance at OD_600nm_ after 7 and 9 days using a spectrophotometer (BioPhotometer Plus, Eppendorf, Germany). *P. citronellolis* P3B5 is able to grow over a wide range of temperatures, i.e. from 16 to 42 °C, with optimal growth ~37 °C (Fig. 1d), however, it is unable to grow at 4 °C.

### Resilience to abiotic factors and antibiotic resistance

On Biolog GenIII plates, *P. citronellolis* P3B5 was able to grow to the same optical density as the positive control in 2 % NaCl solution, to a lower optical density in 5 % NaCl solution, and was unable to grow in 9 % NaCl solution. It was not inhibited by 1 % sodium lactate, rifamycin SV, minocycline, lincomycin, niaproof 4, vancomycin, nalidixic acid, potassium tellurite, and aztreonam. Growth, however not to the same optical density as the positive control, was observed in the presence of fusidic acid, troleandomycin, guanidine HCl, and sodium bromate. No significant growth was detected in presence of d-serine, lithium chloride, and sodium butyrate.

Pseudomonads were previously described to be key players in propagating plasmids, including ABR bearing plasmids, in the phyllosphere [24–28]. Therefore, additional ABR exhibited by *P. citronellolis* P3B5 were determined in antibiotic disc diffusion assays [29]. Out of the tested 32 clinically relevant antibiotics or antibiotic combinations, *P. citronellolis* P3B5 was resistant against ten antibiotics or combinations including the β-lactams cefoxitin, cefpodoxime, cefuroxime, temocillin, cephalothin, cefotaxime, the β-lactam/β-lactamase inhibitor mix amoxicillin/clavulanic acid, and several antibiotics of other classes, i.e. trimethoprim, trimethoprim/sulfamethoxazole, nitrofurantoin, and fosfomycin. *P. citronellolis* P3B5 was not resistant against the following tested antibiotics: cefepime, colistin, tobramycin, gentamycin, amikacin, ciprofloxacin, levofloxacin, sulfonamide, imipenem, and ceftazidime. Resistance was defined based on existing cutoffs, or, when no cutoff was available, as grown completely up to the antibiotic containing disc [29].

## Genome sequencing information

### Genome project history

The organism was selected for sequencing as part of an ongoing project investigating the bacterial diversity on the plant surface (i.e., the phyllobiome) of basil (*Ocimum basilicum* L.). The sequencing project was completed in December 2015; the sequencing data was deposited as a complete genome (one contig representing the complete genome of *P. citronellolis* P3B5) in Genbank under BioProject PRJNA309370, with the accession number CP014158. The genome was sequenced with the Pacific Biosciences RS II platform (Microsynth AG, Switzerland). A summary of the project according to the MIGS version 2.0 [30] is given in Table 2.

**Table 2:** Project information

MIGS ID	Property	Term
MIGS 31	Finishing quality	Complete
MIGS-28	Libraries used	20 Kb PacBio library (BluePippin size selection)
MIGS 29	Sequencing platforms	PacBio RS II
MIGS 31.2	Fold coverage	148×
MIGS 30	Assemblers	HGAP.3
MIGS 32	Gene calling method	Prodigal 2.60
	Locus Tag	PcP3B5
	Genbank ID	CP014158
	GenBank Date of Release	10.03.2016
	BIOPROJECT	PRJNA309370
MIGS 13	Source Material Identifier	P3B5
	Project relevance	phyllosphere, environmental, biochemistry, and bioremediation

### Growth conditions and genomic DNA preparation

To isolate gDNA, cells were grown overnight in terrific broth (12 g l^−1^ tryptone, 24 g l^−1^ yeast extract, 4 ml l^−1^ glycerol, 100 ml l^−1^ 0.17 M KH_2_PO_4_, 0.72 M K_2_HPO_4_) at 30 °C and 250 rpm. gDNA was extracted using the GenElute Bacterial DNA Kit (Sigma-Aldrich, USA) according to the manufacturer’s recommendations. To concentrate gDNA, the primary eluate was precipitated using 5 M ammonium acetate and 100 % ethanol followed by a washing step using 70 % ethanol. The quality and quantity of the extracted DNA was evaluated on a 1.0 % (w/v) agarose gel, by measuring absorption ratios 260_nm_/280_nm_ and 260_nm_/230_nm_, and additionally by performing a Qubit dsDNA GR assay (Life Technologies, USA). To determine the presence of plasmids, a plasmid extraction using the NucleoSpin Plasmid kit (Macherey-Nagel, Germany) was performed according to the manufacturer’s recommendations. However, no plasmids could be detected after running the sample on a 1.0 % (w/v) agarose gel.

### Genome sequencing and assembly

The genome of *P. citronellolis* P3B5 was sequenced with one SMRT cell resulting in a total of 1.35 Gbp. After quality filtering, 98,808 reads with a mean subread read length of 12,474 bp were obtained (Additional file 2: Figure S1). Subsequent *de novo* genome assembly and resequencing steps were performed using PacBio SMRT Portal 2.3.0 [31]. The assembly was generated using the PacBio SMRT Portal protocol RS_HGAP_Assembly.3. Terminal repeats were removed, the genome circularized and its start position was aligned with the dnaA gene using Circlator 1.1.2 [32]. Several rounds of sequence polishing using PacBio SMRT Portal protocol RS_Resequencing.1 with stringent filter criteria (“Minimum Polymerase Read Quality”: 84) were performed, resulting in one 6,951,444-bp contig with an average coverage depth of 148-fold. The assembly did not contain any plasmids and, since virtually all reads were mapped to the genome (97.1 %, other reads were of low quality or chimeric and could still be attributed to the chromosome after manual inspection), provided no evidence for the existence of plasmids. This is in line with the results of the agarose gel electrophoresis analysis described above.

### Genome annotation

Genome annotation was performed using Prokka 1.11.0 [33] with ncRNA search enabled and incorporation of SignalP 4.1 to predict signal peptide cleavage sites [34]. The annotation of selected ORFs annotated as “misc_RNA” by Prokka was manually curated to conform to NCBI requirements. Predicted CDS were then searched against Pfam-A v27.0 [35] and TMHMM 2.0c [36] databases using InterProScan 5.13 [37]. COG categorization was performed by searching predicted CDS against the EggNOG 4.5 database using the *Gammaproteobacteria* specific dataset [38] and subsequent extraction of COG categories.

## Genome properties

**Fig. 3:**
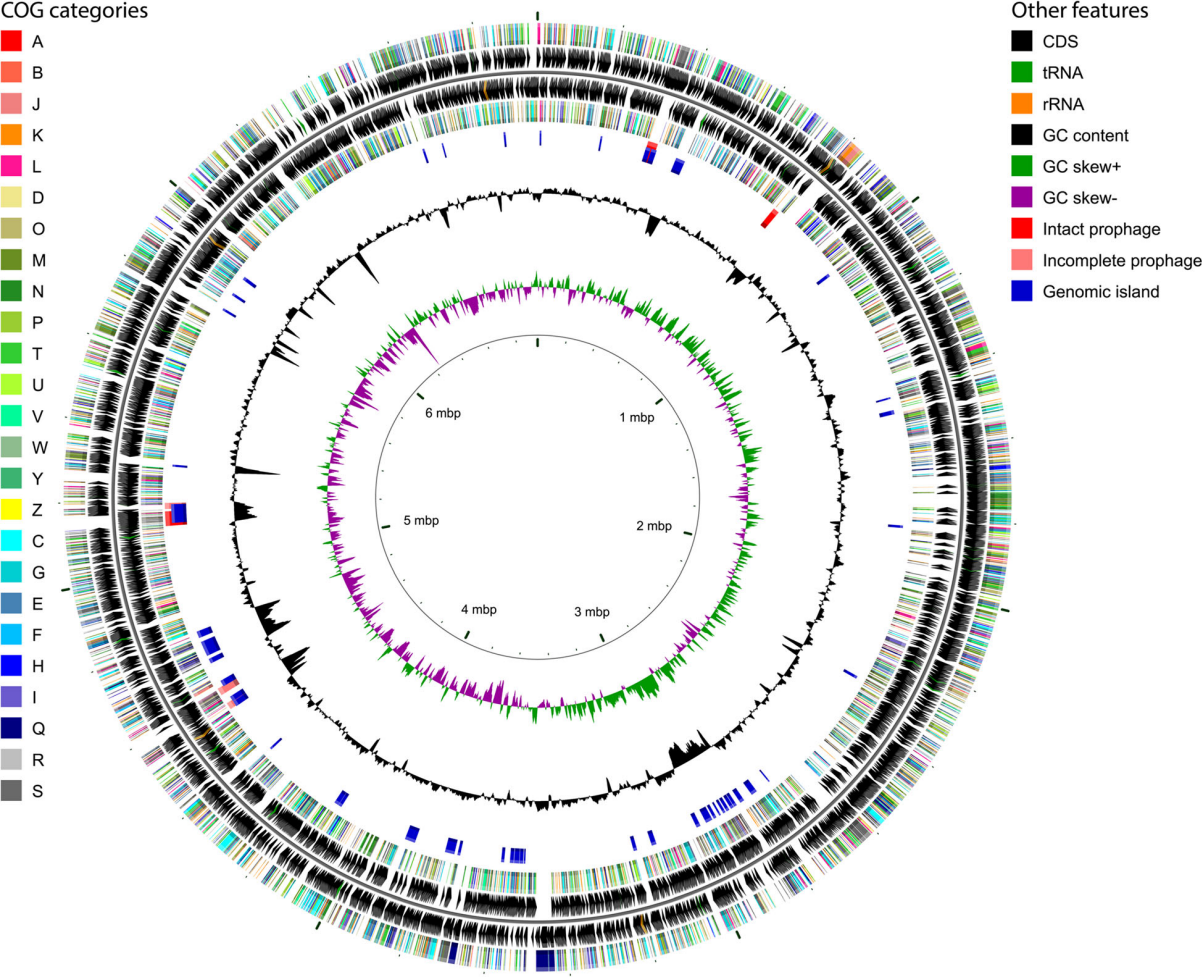
Circular map of the *Pseudomonas citronellolis* P3B5 genome, generated using CGView [70]. Starting from the outmost circle moving inwards, the following tracks are shown: (1) predicted protein coding genes on forward strand colored according to COG categories, (2) CDS (*black*), tRNA (*green*) and rRNA (*orange*) on forward strand, (3) CDS (*black*), tRNA (*green*) and rRNA (*orange*) on reverse strand, (4) predicted protein coding genes on reverse strand colored according to COG categories, (5) Intact prophages (*red*), incomplete prophages (*light red*), and genomic islands (*blue*), (6) GC content (*black*), (7) positive and negative GC skew (*green* and *purple*, respectively) and (8) genome region by mbp

The properties of the complete genome sequence of *P. citronellolis* P3B5 are summarized in Table 3. The average GC content was 67.11 %. Of the 6169 predicted genes, 6071 (98.41 %) were protein CDS of which 4762 genes had a function prediction. Genes without functional prediction by Prokka were annotated as “hypothetical protein”. Two pseudo genes (PcP3B5_29180 and PcP3B5_42810) were predicted by the NCBI annotation pipeline [39] and then incorporated into the Prokka annotation. Of the predicted protein coding genes, 5523 were assigned to COGs of 22 classes (Table 4, Fig. 3). Using the Pfam database, 5242 CDS were assigned to a protein family. Putative transmembrane domains were predicted for 1263 CDS. A signal peptide was predicted for 702 CDS. A total of 96 RNA genes were predicted including 15 rRNA (five complete rRNA operons each comprising a 23S, 16S and 5S rRNA gene), 76 tRNA, 1 tmRNA, and 4 ncRNA genes. Binding sites for cobalamin and thiamine pyrophosphate were predicted by the NCBI annotation pipeline. One CRISPR repeat was predicted by Prokka and further confirmed by CRISPRFinder [40] and PILER-CR [41]. However, no evidence for a Cas protein-coding gene was found in the genome. An analysis of putative prophages using PHAST [42] resulted in six hits in the genome, three of which were designated as *intact* prophages (PcP3B5_02970–PcP3B5_03410; PcP3B5_06890–PcP3B5_07120; PcP3B5_45940–PcP3B5_46520) and the other three as *incomplete* prophages (PcP3B5_40450–PcP3B5_40640; PcP3B5_40870–PcP3B5_41140; PcP3B5_46670–PcP3B5_46960). Genomic islands and ABR genes within genomic islands were predicted using IslandViewer 3 [43]. As references for genomic island analysis, the closely related genomes of *P. knackmussii* B13, *P. denitrificans*ATCC 13867, *P. aeruginosa* PAO1, and *P. stutzeri*DSM 4166 were chosen.

**Table 3:** Genome statistics

Attribute	Value	% of Total
Genome size (bp)	6,951,444	100.00
DNA coding (bp)	6,028,113	86.72
DNA G + C (bp)	4,665,300	67.11
DNA scaffolds	1	100.00
Total genes	6169	100.00
Protein coding genes	6071	98.41
RNA genes	96	1.56
Pseudo genes	2	0.03
Genes in internal clusters	NA	NA
Genes with function prediction	4762	77.19
Genes assigned to COGs	5523	89.53
Genes with Pfam domains	5242	84.97
Genes with signal peptides	702	11.38
Genes with transmembrane helices	1263	20.47
CRISPR repeats	1

**Table 4:** Number of genes associated with general COG functional categories

Code	Value	% age	Description
J	189	3.11	Translation, ribosomal structure and biogenesis
A	1	0.02	RNA processing and modification
K	480	7.91	Transcription
L	180	2.96	Replication, recombination and repair
B	5	0.08	Chromatin structure and dynamics
D	40	0.66	Cell cycle control, Cell division, chromosome partitioning
V	75	1.24	Defense mechanisms
T	286	4.71	Signal transduction mechanisms
M	281	4.63	Cell wall/membrane biogenesis
N	114	1.88	Cell motility
U	79	1.30	Intracellular trafficking and secretion
O	182	3.00	Posttranslational modification, protein turnover, chaperones
C	417	6.87	Energy production and conversion
G	196	3.23	Carbohydrate transport and metabolism
E	489	8.05	Amino acid transport and metabolism
F	112	1.84	Nucleotide transport and metabolism
H	160	2.64	Coenzyme transport and metabolism
I	218	3.59	Lipid transport and metabolism
P	349	5.74	Inorganic ion transport and metabolism
Q	142	2.34	Secondary metabolites biosynthesis, transport and catabolism
R	0	0.00	General function prediction only
S	1528	25.17	Function unknown
-	548	9.03	Not in COGs

The total is based on the total number of protein coding genes in the genome

## Extended insights from the genome sequence

### The *P. citronellolis* P3B5 genome in the light of a phyllosphere associated lifestyle

Plant leaf surfaces, often referred to as phyllosphere, represent an extreme environment to its colonizers which are exposed to largely fluctuating levels of drought, DNA-damaging UV radiation, heat, and oligotrophic nutrient conditions [18]. At the micrometer scale the phyllosphere is very heterogeneous, i.e. habitable sites and nutrient availability is discontinuous, tremendously impacting its colonizers [44, 45].

### Resilience to phyllosphere stress factors

*P. citronellolis* P3B5 did not only survive, but grew at temperatures as high as 44 °C and was also able to grow at moderate temperatures of 16 °C. This ability might represent a fitness advantage on plant leaf surfaces that often feature fluctuating and high temperatures [18]. Adaptation to fluctuating and high temperatures is also reflected in the genome which encodes several heat shock proteins including DnaK (PcP3B5_54370, PcP3B5_56190), GroEL (PcP3B5_12480), and the cold shock proteins CspA_1-CspA_4 (PcP3B5_06040, PcP3B5_17140, PcP3B5_45760, PcP3B5_47880). To counter oxidative stress, *P. citronellolis* P3B5 is equipped with genes encoding proteins known to be involved in oxidative stress reduction such as a manganese-based superoxide dismutase (PcP3B5_11610), a ferrous-based superoxide dismutase (PcP3B5_52370), four catalases (PcP3B5_07860, PcP3B5_23220, PcP3B5_27040, PcP3B5_31600), and several peroxidases (PcP3B5_10150, PcP3B5_32470, PcP3B5_44760). To counter drought stress, *P. citronellolis* P3B5 is equipped with genes encoding for the production of trehalose (PcP3B5_27330) and uptake or production of betaine (PcP3B5_00410–00420, PcP3B5_01390, PcP3B5_01330–01360, PcP3B5_17690, PcP3B5_02060–02090, PcP3B5_25880, PcP3B5_26750–26770, PcP3B5_28710–28740, PcP3B5_28870, PcP3B5_29850, PcP3B5_31730–31770, PcP3B5_34470–34500, PcP3B5_34590, PcP3B5_36730, PcP3B5_43400, PcP3B5_45400, PcP3B5_48640, PcP3B5_50450, PcP3B5_58450–58430, PcP3B5_60970, PcP3B5_61100, PcP3B5_61120–61140, PcP3B5_61750, PcP3B5_62040), two osmoprotectants. Notably, no other representative pseudomonad currently found in the SEED database [46] features as many betaine related uptake systems, indicating the importance of this osmoprotectant for the lifestyle of *P. citronellolis* P3B5. This suggests a phyllosphere adapted lifestyle which requires the ability to survive and thrive under constant drought stress. The phyllosphere has furthermore been described to be iron limited [47], therefore, the acquisition of and competition for iron is crucial on plant leaves. The *P. citronellolis* P3B5 genome, as is common in pseudomonads, encodes for several genes involved in the acquisition of iron by the production and uptake of siderophores.

### Potential resource utilization on plant surfaces

*P. citronellolis* P3B5 is equipped for the acquisition and utilization of many nutrients that are available in the phyllosphere, especially different carbohydrates and amino acids [48, 49]. Genes encoding for ABC transporters for amino acids found on plant surfaces (arginine, cysteine, glycine, histidine, methionine, proline), dipeptides, oligopeptides, branched amino acids, putrescine, spermidine, lipopolysaccharides, glucose, and nitrate. Furthermore, genes encoding for a phosphotransferase uptake system for fructose are present, as well as major facilitator superfamily protein sugar transporter and other sugar transporters. To predict the ability of *P. citronellolis* P3B5 to metabolize various compounds, RAST [50] and modelSEED were employed (http://modelseed.org) [46, 51, 52]. Genetic evidence for the metabolic utilization of glucose, fructose, and sucrose, the three most prevalent photosynthates found on leaves, was found [48, 53]. Furthermore, genes predicted to be involved in the degradation of short organic acids which can be found on plant leaves were detected, e.g. citrate, fumarate, glycolate, malate, maleate, pyruvate, succinate, and tartrate [48].

Recently, the soil-borne *Pseudomonas aeruginosa* SJTD-1, a strain phylogenetically related to *P. citronellolis*, was shown to be able to degrade medium and long chain alkanes from n-tetradecane (C_14_) to n-tetracosane (C_24_) due to the protein alkane monooxygenase [54]. The presence of two AlkB-like alkane monooxygenase genes in the *P. citronellolis* P3B5 genome (PcP3B5_23990, PcP3B5_31900) might enable it to nutritionally access long and very-long chain alkanes available on plant leaves, thereby gaining a fitness advantage over other phyllosphere colonizers. Furthermore, *P. citronellolis* P3B5 contains two putative AlmA-like monooxygenases (PcP3B5_03500, PcP3B5_37240), which would allow the degradation of very long chain alkanes (>C_30_) [55]. *P. citronellolis* P3B5 was isolated from herbs that are rich in aromatic oils, such as terpenes [12]. Previously it has been shown that *P. citronellolis* is able to degrade terpenes such as citronellol [11] and a corresponding gene cluster (PcP3B5_19950–20010) was also detected in *P. citronellolis* P3B5, which may be responsible for enabling this species to colonize the phyllosphere of aromatic herbs.

### Biotic relationship to the plant host

*P. citronellolis* P3B5 and other *P. citronellolis* isolates were isolated from healthy plant material and plants from the same field plot did not exhibit disease symptoms during the course of the survey. Therefore, it is unlikely that *P. citronellolis* is a plant pathogen. It is unclear if the strain is able to confer a health promoting effect on its plant host, thus it is prudent to classify it as tritagonist, an organism whose biotic relationship to its host is thus far undescribed [56]. However, the *P. citronellolis* P3B5 genome contains genes whose products are involved in the biosynthesis of indole-3-acetic acid (PcP3B5_05210–05220, PcP3B5_17810, PcP3B5_07120–07140), a compound often found to be produced by bacteria associated with plants [18]. Indole-3-acetic acid is a plant hormone of the auxin class which induces plant cell elongation and division, which leads to an increase of local available nutrients [57]. This indicates that *P. citronellolis* P3B5 is able to impact on the plant host.

### Antibiotic resistance and possible horizontal gene transfer of resistance genes

The genomic data broadly supports the phenomenological antibiotic screens described above, the respective putative ABR genes and loci are summarized in Table 5. Many of the tested compounds were β-lactam antibiotics of different generations. The P3B5 genome contains genes encoding for six predicted β-lactamases, providing resistances against many β-lactam antibiotics, including a class C β-lactamase conferring resistance to cephalosporin antibiotics and putatively to the monobactam aztreonam. Furthermore, the genome contains genes encoding for efflux pumps that provide resistances to other antibiotics, such as trimethoprim.

**Table 5:** Identified antibiotic resistances and their putative genetic background

Antibiotic	Class	Encoded resistance genes; locus
amoxicillin/clavulanic acid	β-lactam + β-lactamase inhibitor	metallo-β-lactamase, PcP3B5_32180 class A β-lactamase, PcP3B5_44400 metallo-β-lactamase, PcP3B5_46940 metallo-β-lactamase, PcP3B5_47300 class C β-lactamase, PcP3B5_53150
cefotaxime	β-lactam/third generation cephalosporin	
cefoxitin	β-lactam/second generation cephalosporin	
cefpodoxime	β-lactam/third generation cephalosporin	
cefuroxime	β-lactam/second generation cephalosporin	
cephalothin	β-lactam/second generation cephalosporin	
temocillin	β-lactam/β-lactamase-resistant penicillin	
aztreonam	monobactam antibiotic	class C β-lactamase, PcP3B5_53150
lincosamide		intrinsic resistance
vancomycin		intrinsic resistance
minocycline	tetracycline antibiotic	tetA, PcP3B5_30660
rifamycin SV	rifamycin	intrinsic resistance, no resistance genes detected
trimethoprim	sulfonamide antibiotic	multidrug efflux pump OprM1-5; PcP3B5_05670, PcP3B5_32430, PcP3B5_35160, PcP3B5_36300, PcP3B5_37480
trimethoprim/sulfamethoxazole	dihydrofolate reductase inhibitor/sulfonamide antibiotic	
nitrofurantoin	nitrofuran derivative	intrinsic resistance and vanX; PcP3B5_55530
fosfomycin	*N*-acetylmuramic acid synthesis inhibition	fosA; PcP3B5_27860

To further analyze the potential of described ABR genes to be mobilized by horizontal gene transfer, it was investigated if they are located on mobile genetic elements, i.e. prophages, transposons, and genomic islands. None of the six putative prophage related genomic loci or the predicted genomic islands contained genes encoding for ABR. Furthermore, after close inspection of flanking regions 30 kbp up- and downstream of resistance genes, no evidence for functional transposable elements could be detected.

### Bioremediation potential

The capability of *P. citronellolis* P3B5 to degrade long-chain alkanes and terpenes in combination with a predicted resilience to many environmental stresses make it an ideal candidate for future bioremediation applications. Also, a recent study highlighted the ability of a *P. citronellolis* isolate to be able to degrade low density polyethylene [58] and, moreover, *P. citronellolis* P3B5 is closely related to *P. knackmussi*, which is known for its potential in biodegradation of chloroaromatic compounds [59]. This further hints towards a, yet to be further investigated, broad biodegradation potential of *P. citronellolis*. Its preferred niche on plant surfaces can potentially be further exploited for so-called “phyllo-remediation” approaches during which bacteria are employed to degrade organic-pollutants on plant foliage [60, 61].

## Conclusions

We provide a comprehensive insight into the genome and biochemical properties of the environmentally and biotechnologically interesting species *P. citronellolis*. Furthermore, we provide an interpretation of its environmental life-style on plant leaves from a genomic perspective. The *P. citronellolis* P3B5 genome encodes genes that should enable it to degrade long- and very long-chain alkanes, and terpenes. This metabolic capability, in combination with its stress-resilience and phyllosphere lifestyle, makes the organism an intriguing candidate for phyllo-remediation based bioremediation approaches. Resistance to several ABs was observed and several ABR genes were detected, but no evidence for the potential of ABR gene mobilization could be found. The availability of the complete genome sequence of *P. citronellolis* will facilitate future comparative genomics approaches of the phylogenetically broad genus *Pseudomonas*, which is currently understudied and biased towards pathogenic species. To fully appreciate the genetic diversity of the genus even more species should be complete genome sequenced in the future. This will also enable hypothesis-driven research on the difference between pathogenic and non-pathogenic species.

## Additional files

Additional file 1: Table S1. Accession numbers of representative *Pseudomonas* genomes used to generate the phylogenetical trees in Fig. 2 ﻿and Additional file 2: Figure S2. (DOCX 70 kb) Additional file 2: Figure S1. Subread length distribution after sequencing. **Figure S2**. Phylogenetic tree of the genus *Pseudomonas* highlighting the position of *P. citronellolis* P3B5 relative to other representative *Pseudomonas* species. *Xanthomonas campestris* pv. *campestris* ATCC 33913 was chosen as outgroup. The tree is based on a MLSA using four housekeeping gene sequences (16S rRNA, gyrB, rpoB, rpoD). The bar represents the number of base substitutions per site. The percentage of replicate trees in which associated taxa clustered in the bootstrap test with 1000 replicates are shown next to the respective branches. Accession numbers of the used strains are reported in Additional file 1: Table S1. (DOCX 295 kb)

## Abbreviations

ABR: Antibiotic resistance
gDNA: genomic DNA
MALDI: Matrix-assisted laser desorption/ionization
MALDI-TOF: MALDI-time of flight
MLSA: Multilocus sequence analysis
TBX agar: Tryptone Bile X-Glucuronide agar
