# Supplementary material for: Complete genome sequence of Pseudomonas citronellolis P3B5, a candidate for microbial phyllo-remediation of hydrocarbon-contaminated sites
Source: Stand Genomic Sci. 2016 Sep 26;11:75. doi: 10.1186/s40793-016-0190-6 (PMC5037603; doi:10.1186/s40793-016-0190-6)
Supplement: Additional file 1: Table S1. — Accession numbers of representative Pseudomonas genomes used to generate the phylogenetical trees in Fig. 2 ﻿and Additional file 2: Figure S2. (DOCX 70 kb) [file 40793_2016_190_MOESM1_ESM.docx]

**Supplemental table 1.** Accession numbers of representative *Pseudomonas* genomes used to generate the phylogenetical tree in figure 2.

| **Strain name** | Accession number | Type strain |
| --- | --- | --- |
| *P. aeruginosa* PAO1 | AE004091 | no |
| *P. alkylphenolia* KL28 | CP009048 | yes |
| *P. balearica* DSM 6083 | CP007511 | yes |
| *P. brassicacearum* subsp. *brassicacearum* NFM421 | CP002585 | no |
| *P. chlororaphis* PA23 | CP008696 | no |
| *P. cichorii* JBC1 | CP007039 | no |
| *P. citronellolis P3B5* | CP014158 | no |
| *P. cremoricolorata* ND07 | CP009455 | no |
| *P. denitrificans* ATCC 13867 | CP004143 | no |
| *P. entomophila* L48 | CT573326 | yes |
| *P. fluorescens* Pf0-1 | CP000094 | no |
| *P. fulva* 12-X | CP002727 | no |
| *P. knackmussii* B13 | HG322950 | yes |
| *P. mandelii* JR-1 | CP005960 | no |
| *P. mendocina* NK-01 | CP002620 | no |
| *P. monteilii* SB3101 | CP006979 | no |
| *P. mosselii* SJ10 | CP009365 | no |
| *P. parafulva* CRS01-1 | CP009747 | no |
| *P. plecoglossicida* NyZ12 | CP010359 | no |
| *P. poae* RE*1-1-14 | CP004045 | no |
| *P. protegens* Pf-5 | CP000076 | yes |
| *P. putida* KT2440 | AE015451 | no |
| *P. resinovorans* NBRC 106553 | AP013068 | no |
| *P. rhizosphaerae* DSM 16299 | CP009533 | no |
| *P. stutzeri* ATCC 17588 | CP002881 | yes |
| *P. syringae* pv. *phaseolicola* 1448A | CP000058 | no |
| *P. syringae* pv. *syringae* B728a | CP000075 | no |
| *P. syringae* pv. *tomato* DC3000 | AE016853 | no |
| *P. trivialis* IHBB745 | CP011507 | no |
| *X. campestris* pv. *campestris* ATCC 33913 | NC_003902 | no |
