# Supplementary material for: Complete genome sequence of Pseudomonas citronellolis P3B5, a candidate for microbial phyllo-remediation of hydrocarbon-contaminated sites
Source: Stand Genomic Sci. 2016 Sep 26;11:75. doi: 10.1186/s40793-016-0190-6 (PMC5037603; doi:10.1186/s40793-016-0190-6)
Supplement: Additional file 2: Figure S1. — Subread length distribution after sequencing. Figure S2. Phylogenetic tree of the genus Pseudomonas highlighting the position of P. citronellolis P3B5 relative to other representative Pseudomonas species. Xanthomonas campestris pv. campestris ATCC 33913 was chosen as outgroup. The tree is based on a MLSA using four housekeeping gene sequences (16S rRNA, gyrB, rpoB, rpoD). The bar represents the number of base substitutions per site. The percentage of replicate trees in which associated taxa clustered in the bootstrap test with 1000 replicates are shown next to the respective branches. Accession numbers of the used strains are reported in Additional file 1: Table S1. (DOCX 295 kb) [file 40793_2016_190_MOESM2_ESM.docx]

**Supplemental figure 1** Subread length distribution after sequencing

**Supplemental figure 2.** Phylogenetic tree of the genus *Pseudomonas* highlighting the position of *P. citronellolis* P3B5 relative to other representative *Pseudomonas* species. *Xanthomonas campestris* pv. *campestris* ATCC 33913 was chosen as outgroup. The tree is based on a MLSA [1] using four housekeeping gene sequences (16S rRNA, gyrB, rpoB, rpoD). The bar represents the number of base substitutions per site. The percentage of replicate trees in which associated taxa clustered in the bootstrap test with 1000 replicates are shown next to the respective branches. Accession numbers of the used strains are reported in supplemental table 1

1. Glaeser SP, Kämpfer P: **Multilocus sequence analysis (MLSA) in prokaryotic taxonomy.** *Systematic and Applied Microbiology* 2015, **38:**237-245.
